# Further evidence for an anti-inflammatory role of artesunate in experimental cerebral malaria

**DOI:** 10.1186/1475-2875-12-388

**Published:** 2013-11-02

**Authors:** Aline S Miranda, Fátima Brant, Natália P Rocha, Daniel Cisalpino, David H Rodrigues, Danielle G Souza, Fabiana S Machado, Milene A Rachid, Antônio L Teixeira Jr, Alline C Campos

**Affiliations:** 1 Programme in Health Sciences: Infectious Diseases and Tropical Medicine, School of Medicine, Federal University of Minas Gerais, Belo Horizonte, MG, Brazil; 2 Laboratório de Imunofarmacologia, Department of Biochemistry and Immunology, Institute of Biological Sciences, Federal University of Minas Gerais, Av. Antônio Carlos, 6627, Pampulha, Belo Horizonte 31270-901, Brazil; 3 Department of Microbiology, Institute of Biological Sciences, Federal University of Minas Gerais, Belo Horizonte, MG, Brazil; 4 Department of Pathology, Institute of Biological Sciences, Federal University of Minas Gerais, Belo Horizonte, MG, Brazil; 5 Laboratory of Medical Investigation, School of Medicine, Federal University of Minas Gerais, Belo Horizonte, MG, Brazil

**Keywords:** Malaria, Cerebral malaria, Memory impairment, Artesunate, Cytokines, Neuroinflammation

## Abstract

**Background:**

Cerebral malaria (CM) is a clinical syndrome resulting from *Plasmodium falciparum* infection. A wide range of clinical manifestations follow the disease including cognitive dysfunction, seizures and coma. CM pathogenesis remains incompletely understood and without treatment this condition is invariably fatal. Artesunate has been accepted as the most effective drug for treating severe malaria. Besides its antiparasitic activity, an anti-inflammatory property has also been reported. In the current study, the immunomodulatory role of artesunate was investigated using a *Plasmodium berghei* ANKA model of CM, trough evaluation of behavioural changes and cytokines expression in hippocampus and in frontal cortex.

**Methods:**

C57Bl/6 mice were infected with *P. berghei* by intraperitoneal route, using a standardized inoculation of 10^6^ parasitized erythrocytes. Memory function was evaluated using the step-down inhibitory avoidance test. The mRNA expression of IFN-γ, IL-1β, IL-6 and TNF in the frontal cortex and hippocampus of control and infected mice on day 5 post-infection were estimated by quantitative real time PCR. *Plasmodium berghei* -infected mice also received intraperitoneally a single dose of artesunate (32 mg/kg) on day 4 post-infection, and 24 hours after treatment behavioural and immunological analysis were performed. The protein levels of cytokines IL-2, IL-6, IL-10, IL-17, IFN-γ, TNF in the serum, frontal cortex and hippocampus of controls and *P. berghei* -infected mice treated or not treated with artesunate were determined using a cytometric bead array (CBA) kit. The survival and neurological symptoms of CM were also registered.

**Results:**

CM mice presented a significant impairment of aversive memory compared to controls on day 5 post-infection. A higher mRNA expression of pro-inflammatory cytokines was found in the hippocampus and frontal cortex of infected mice. A single dose of artesunate was also able to decrease the expression of inflammatory cytokines in the hippocampus and frontal cortex of *P. berghei*-infected mice. In parallel, a significant improvement in neurological symptoms and survival were observed in artesunate treated mice.

**Conclusions:**

In summary, the current study provided further evidence that CM affects key brain areas related to cognition process. In addition, different patterns of cytokine expression during the course of CM could be modulated by a single administration of the anti-malarial artesunate.

## Background

Cerebral malaria (CM) is a potentially reversible, diffuse encephalopathy characterized mainly by the presence of asexual forms of *Plasmodium falciparum* parasites in the peripheral blood smears, and altered level of consciousness in the absence of other causes of encephalopathy, especially meningitis and viral encephalitis [1]. This condition accounts for approximately 80% of all fatal cases of malaria, being the leading cause of hospitalization and mortality of children under five years of age in sub-Saharan Africa [2,3]. A series of residual symptoms may follow the resolution of CM including cognitive, behavioural and motor changes [4]. Moreover, a significant social, economic and educational burden has been reported in endemic areas of malaria [5].

Although vascular, immunological and metabolic changes have been implicated in CM pathogenesis, it remains largely unknown [6]. The obstruction of cerebral microvasculature by sequestered infected red blood cells and an exacerbation in host inflammatory response are the two main non-exclusive theories widely accepted to explain this pathological process [7].

The release of pro-inflammatory mediators, including tumor necrosis factor (TNF) and interleukin (IL)-1, and immune cells infiltration in the brain parenchyma have been associated with cognitive and behavioural alterations during the acute phase of experimental CM [8-10]. However, it was only recently that a study conducted by Linares *et al*. [11] suggested that the immune response evoked by CM can specifically affect key brain areas involved in the control of cognition and mnemonic process, such as the hippocampus and the frontal cortex.

Without treatment CM invariably leads to death. Emergency management is based on the correction of metabolic states, re-establishment of vital physiological functions severely affected by CM and the administration of an effective anti-parasitic drug [12].

Artesunate, a semi-synthetic derivative of artemisinin, has been accepted as the most effective and safe drug for treating severe and chloroquine-resistant malaria [13]. Besides its antiparasitic activity, artesunate has shown to exhibit a putative anti-inflammatory profile in different inflammatory models, including sepsis, arthritis, systemic lupus erythematous and allergic asthma [14-18]. Clemmer *et al*. [19] also demonstrated that artesunate was effective in rescuing mice in a late-stage CM and that artemether, another compound derivative of artemisinin, decreases leukocyte accumulation in brain vessels. However, no previous study has investigated the immunomodulatory properties of artesunate in the central nervous system (CNS) during the progression of CM.

The present study aimed to characterize the role of inflammatory mediated process within the hippocampus and frontal cortex in the behavioural changes (memory impairment and sickness behaviour) induced by CM. In addition, the putative immunomodulatory effect of a single dose of artesunate was also investigated in the experimental model of CM.

## Methods

### Ethics statement

This study was carried out in strict accordance with the Brazilian Government’s ethical and animal experiments regulations. The experimental protocol was approved by the Committee on the Ethics of Animal Experiments of the Universidade Federal de Minas Gerais (CETEA/UFMG, Permit Protocol Number 105/09). All tissue-collected procedures was performed under ketamine/xylazine anesthesia and all efforts were made to minimize animal suffering.

### Animals

Female C57BL/6 mice (20-25 g), aged six to eight weeks, were obtained from Animal Care Facilities of the Institute of Biological Sciences, Federal University of Minas Gerais (ICB-UFMG), Belo Horizonte, Brazil. The animals were housed in groups of six mice per cage in a room controlled temperature (24°C) with food and water *ad libitum*. Experiments were performed on day 5 post-infection (pi), when mice infected with *Plasmodium berghei* strain ANKA develop brain inflammation without motor impairment.

### Parasite, experimental infection and artesunate treatment

Due to the high degree of reproducibility, easily manageable characteristics and development of histopathological and neurological signs typical of human CM, the murine model using the *Plasmodium berghei* strain ANKA has been widely used to better understand this condition [20]. In the present study, blood stages of *P. berghei* strain ANKA constitutively expressing green fluorescent protein (*P. berghei* ANKA-GFP) (15cy1 clone), kindly provided by Dr Claudio Marinho (Universidade de São Paulo), were stored in liquid nitrogen [19]. Mice were infected intraperitoneally (ip) with 10^6^*P. berghei*-infected red blood cells suspended in 0.2 mL PBS [21]. Artesunate (Sigma, St. Louis, MO, USA) was dissolved in 5% sodium bicarbonate in saline and a single dose (32 mg/kg) was administered ip in the morning of the fourth day pi [19,22]. Control animals received the same volume of vehicle.

The percentage of parasitaemia was determined by flow cytometry. Briefly, a drop of blood from the tail was collected directly into 2 ml of PBS for flow cytometry analysis. Each sample was run on a FACScalibur (Becton Dickinson, San Jose, CA, USA) flow cytometer with a 488 nm argon laser and DIVA software (Becton Dickinson, San Jose, CA, USA). Erythrocytes were identified on the basis of their specific forward (FSC) and side (SSC) light-scattering properties and a total of 100,000 events were counted for each sample. Mice were observed daily for parasitaemia, body weight, survival, and clinical neurological signs of CM (i e, ataxia, paralysis and coma).

### Step-down inhibitory avoidance test

The step-down inhibitory avoidance test was performed to assess short- and long-term aversive memory on day 5 and 6 post-infection (control and infected mice; n = 10 per group) as previously described by Reis *et al*. [23]. Briefly, in the training trial, animals were placed on the platform and their latency to step down on the grid with all four paws was measured. Immediately after the stepping down on the grid, the animals received a single mild foot shock (0.4 mA, 2.0 seconds). A retention test trial was performed 1.5 hour and 24 hours after the training section. The results were expressed as latency period to step down the platform, with a cut-off at 180 seconds. All tests were performed by the same investigator who was blinded to the animal status (control or infected).

### Open field test

The open field test was performed as previously described by Barichello *et al.*[24] and conducted in order to evaluate the locomotor and exploratory activities on day 5 and 6 pi of control and infected mice (n = 5 per group). The apparatus is a 30 cm × 30 cm circular open field surrounded by 30 cm-high walls made of transparent plexiglass (Insight®, São Paulo, Brazil). The floor of the open field is divided in 12 rectangles by black lines. Briefly, animals were gently placed on the left rear quadrant, and then they were allowed to explore the arena for 5 minutes. The number of crossings (the number of times that animals crossed the black lines) and rearings (the exploration behaviour observed in rats subjected to a new environment) were recorded. Behavioural tests were performed by the same investigator who was blinded to the animal status (control or infected).

### Rapid murine coma and behaviour scale (RMCBS)

Behavioural and functional parameters were evaluated using the RMCBS protocol as previously described by Carrol *et al*. [25]. The RMCBS is a quantitative and objective scale that enables an investigator to rapidly follow up the course of CM. The subjects were labelled as affected or not, and the levels of illness are correlated with neuropathological injury. This method intended to imitate the situation in the field and attempted to bring the animal model closer to the human disease [25].

The RMCBS consists of ten parameters (gait, balance, motor performance, body position, limb strength, touch scape, pinna reflex, toe pinch, aggression and grooming) based on the components of the SHIRPA (SmithKline/Harwell/Imperial College/Royal Hospital/Phenotype Assessment) score [26]. Each item is scored from zero as the lowest, to two as the highest, with a maximum total score of 20. The procedure was carried out on day 3 until day 5 pi. On day 5 pi the RMCBS were performed approximately 24 hours after artesunate treatment. A total of five animals per group were used. After the test all animals were sacrificed under deep anesthesia by ip injection of a mixture of Ketamine (150 mg/kg, Laboratório Cristália, Brazil) and Xylazine (10 mg/kg, Rompun®, Bayer, Germany), decapitated, frontal cortex and hippocampal dissected and serum collected for posterior cytokine expression assessment.

### Hippocampal and frontal cortex mRNA expression of cytokines

The mRNA expression of IFN-γ, IL-1β, IL-6 and TNF in the hippocampus and frontal cortex of *P. berghei*-infected mice and matched controls (n = 5 per group) were estimated by quantitative real time PCR (polymerase chain reaction) at day 5 pi. RNA isolation was performed using Illustra RNAspin Mini RNA Isolation Kit (GE Healthcare, Little Chalfont, Buckinghamshire, UK). The RNA obtained was re-suspended in diethyl pyrocarbonate-treated water and stocked at -70°C until use. Reverse transcription was performed using 2 μg of total RNA, 200 U of reverse transcriptase, RT buffer 5 × (4 μl), 10 mM dNTPs (1 μl), RNAsin 10,000 U (0.2 μl) and oligo dT 15 50 μM (1.0 μl) in a final reaction volume of 20 μl. Resultant cDNA was used for qPCR. Real-time RT-PCR was performed on an ABI PRISM 7900 sequence-detection system (Applied Biosystems, CA, USA) by using SYBR Green PCR Master Mix (Applied Biosystems, CA, USA) after a reverse transcription reaction of 2 μg of total RNA by using M-MLV reverse transcriptase (Promega, Madison, Wisconsin-WI, USA). Specific primers were designed using Primer Express software and synthesized by integrated DNA technologies (IDT). The relative levels of gene expression were determined by the ΔΔ Cycle threshold method as described by the manufacturer, in which data for each sample is normalized to 18S expression and data were shown as fold increase over the negative control (non-infected) group. Specific primers sequences are described in Table 1.

**Table 1:** Oligonucleotide primers for real-time quantitative PCR (SYBR)

**Primer sequences**		
	**Forward**	**Reverse**
IFN-γ	CAG GAT CCT TTG GAC CCT CTG AC	GGC AGA ATT AAG CTT ATT GGG AC
TNF-α	ACG GCA TGG ATC TCA AAG AC	AGA TAG CAA ATC GGC TGA CG
IL-1β	CTA CAG GCT CCG AGA TGA ACA AC	TCC ATT GAG GTG GAG AGC TTT C
IL-6	TTC CAT CCA GTT GCC TTC TTG	TTG GGA GTG GTA TCC TCT GTG A
18S	CGT TCC ACC AAC TAA GAA CG	CTC AAC ACG GGA AAC CTC AC

### Cytometric bead array (CBA) and enzyme-linked immunosorbent assay (ELISA) analyses of cytokines

Hippocampal and frontal cortex tissues of controls (n = 5 per group) and *P. berghei*-infected mice treated or not treated (n = 5 per group) with a single dose of artesunate were carefully removed on day 5 pi and homogenized in a PBS-buffer extraction solution containing a protease-inhibitor cocktail. Lysates were centrifuged at 13,000 g for 10 minutes at 4°C and quantified using the Bradford assay reagent from Bio-Rad (Hercules, CA, USA). Analyses of serum and brain cytokine levels were determined using a mouse Th1/Th2/Th17 cytometric bead array kit (CBA; BD Biosciences, San Diego, CA, USA) and analysed on a FACSCalibur flow cytometer (Becton Dickinson, San Jose, CA, USA). The following cytokines were measured: interleukin-2 (IL-2), interleukin-6 (IL-6), interleukin-10 (IL-10), interleukin-17 (IL-17), Interferon-γ (IFN-γ) and tumor necrosis factor-α (TNF). The concentration of the cytokine IL-1β was determined by ELISA (R&D Systems, Minneapolis, MN, USA) in accordance with manufacturer’s instructions.

### Data analysis and statistical evaluation

Results obtained were presented as mean ± standard error of the mean (SEM). All data were tested for normality (Kolmorov-Smirnov’s test) and homogeneity of variances (Levene’s test). For variables normally distributed and with compliance of homogeneity of variances, differences were compared by using Student’s t test, analysis of variance (ANOVA) or Two-way ANOVA. Duncan’s post-test was used as needed for multiple comparisons. In the case of variables not normally distributed, differences were analysed by Kruskal-Wallis non-parametric test. When significant effects were detected by this latter test, post-hoc comparisons were performed using the Mann-Whitney U test. Differences between lethality curves were calculated using the Log rank test. The significance level was set at the level of p < 0.05. Statistical analyses were performed using Prism 4 software (GraphPad, La Jolla, CA, USA) and SPSS software version 17.0 (SPSS Inc, Chicago, IL, USA).

## Results

### *Plasmodium berghei*-infected mice exhibited short-term aversive memory impairment

**Figure 1:**
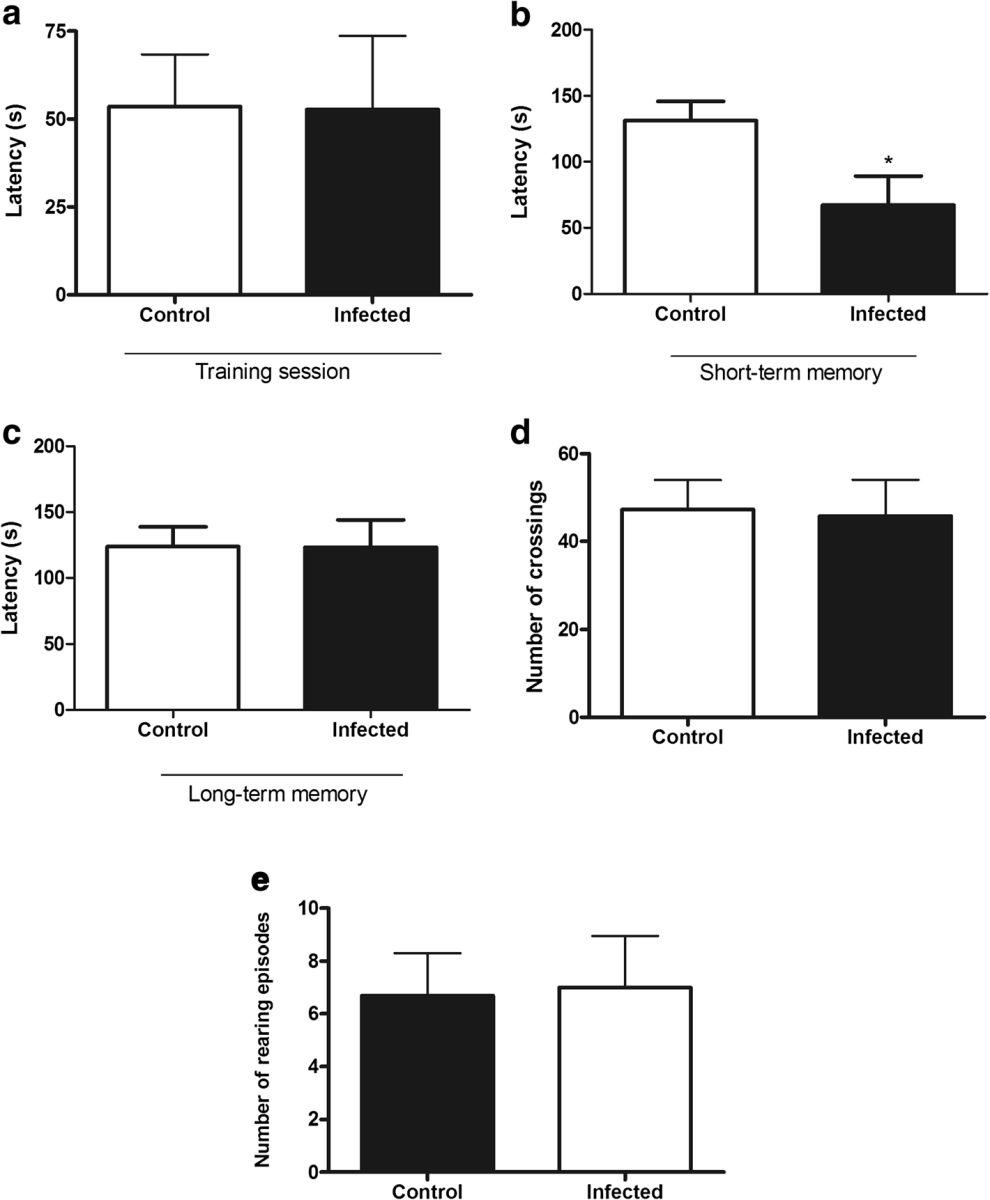
**Short-term aversive memory impairment without locomotor and exploratory changes following *Plasmodium berghei *ANKA infection.** Aversive memory, locomotor and exploratory activities measurement of *P. berghei*-infected mice on day 5 and 6 post-infection (pi) and of control group in the step-down inhibitory avoidance test **(a-c)** and in the open field **(d-e)**. **(a)** Latency in seconds during training session (5 pi) in the step-down inhibitory avoidance test; **(b)** Short-term memory analysed 1.5 hours after training session in the step-down inhibitory avoidance test; **(c)** Long-term memory analysed 24 hours (6 pi) after training session in the step-down inhibitory avoidance test; **(d)** number of crossings and **(e)** number of rearing episodes analysed on day 5 post-infection in the open field. Results are expressed as mean ± SEM and are representative of two independent experiments. Asterisk(s) indicate statistical differences, *p < 0.05.

Figure 1 shows the effect of *P. berghei* infection in the step-down inhibitory avoidance test and open field test. No difference in the step-down latency between *P. berghei*-infected mice and controls was found in the training session (Figure 1a). In the test session performed 1.5 hours after the training to evaluate the short-term aversive memory, infected mice presented a significant decrease in the step-down latency (Figure 1b; p = 0.03). No difference was found in the long-term aversive memory analysed 24 hours (day 6 pi) after training session (Figure 1c; p = 0.99).

In the open field test, there were no significant differences in the number of crossings (Figure 1d; p = 0.89) and rearings (Figure 1e; p = 0.91) in the *P. berghei*-infected group compared to the controls on day 5 pi, indicating no difference in motor and exploratory activities, respectively. However, on day 6 pi, the period when the long-term memory was assessed, *P. berghei*-infected mice presented significant deficits in motor (p = 0.001) and exploratory (p = 0.002) activities (data not shown).

### Inflammatory changes in hippocampus and frontal cortex of *P. berghei*-infected mice, key areas related to memory and learning

In order to evaluate inflammatory parameters in specific regions well known to participate in memory formation and consolidation, the mRNA expression of IFN-γ, TNF, IL-1β and IL-6 and the protein levels of IFN-γ, TNF, IL-1β, IL-2, IL-4, IL-6, IL-10 and IL-17 was analyzed in hippocampus and frontal cortex of controls and *P. berghei*-infected mice on day 5 pi.

**Figure 2:**
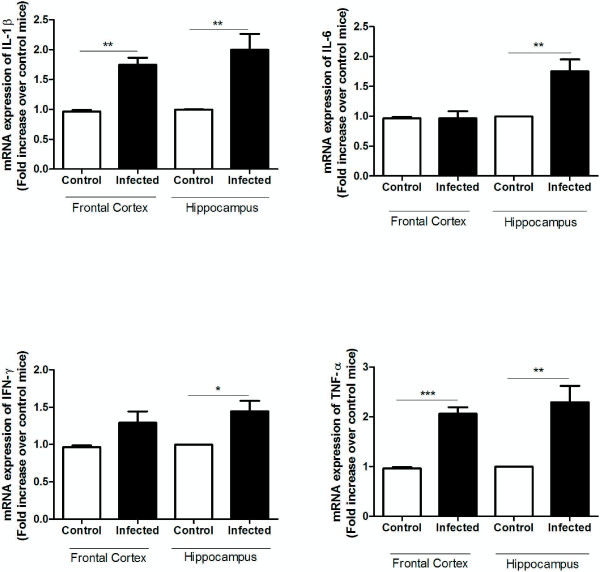
**Frontal cortex and hippocampus mRNA expression of pro-inflammatory cytokines following *Plasmodium berghei *ANKA infection.** C57BL/6 mice were intraperitoneally infected with 10^6^ parasitized erythrocytes and control (non-infected) animals received the same volume of PBS (n = 5 *per group*). On day 5 post-infection (5 dpi) frontal cortex and hippocampus were collected for real time PCR analysis. Results use arbitrary units for the ratio of the target gene mRNA to the endogenous control, eukaryotic 18SmRNA. Statistical significance: *p < 0.05, **p < 0.01, ***p < 0.001.

The mRNA expression of TNF and IL-1β was significantly increased in the frontal cortex and hippocampus of *P. berghei*-infected mice compared to controls. A higher mRNA expression of IFN-γ and IL-6 was only observed in the hippocampus of *P. berghei*-infected mice (Figure 2).

**Figure 3:**
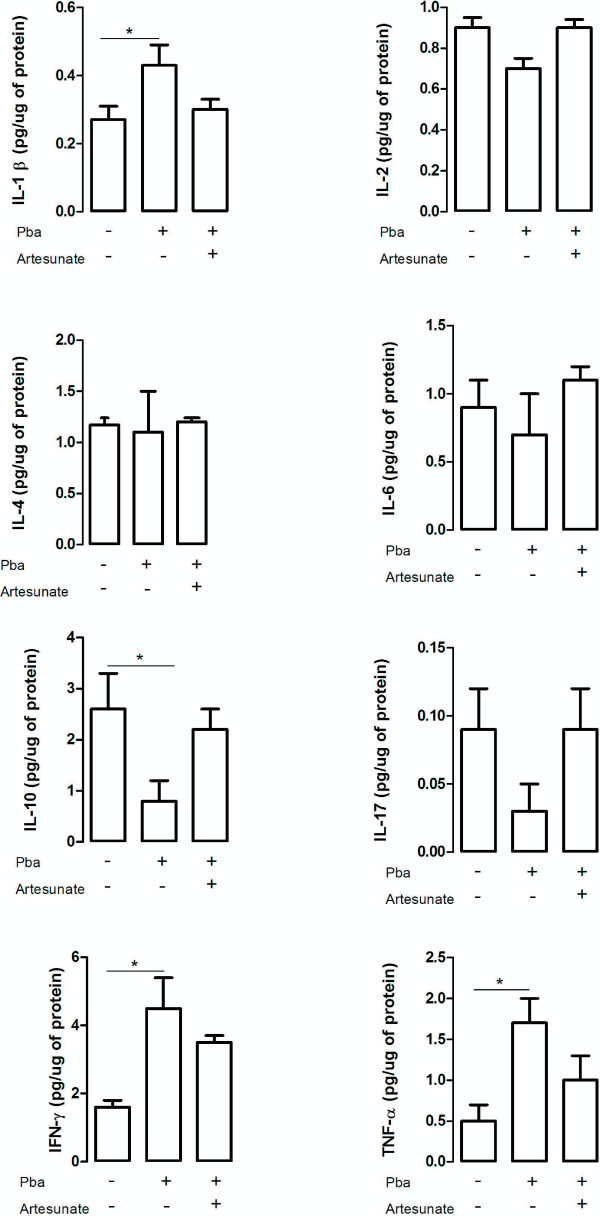
**Cytokine levels in the frontal cortex of controls and infected mice following artesunate treatment.** C57BL/6 mice were intraperitoneally infected with 106 parasitized erythrocytes and control (non-infected) animals received the same volume of PBS (n = 5 *per group*). On day 4 post-infection *P. berghei*-infected (mice received a single dose of artesunate (ip 32 mg/kg). The frontal cortex was collected on day 5 post-infection, homogenized and IL-1β expression was measured by ELISA while IFN-γ, TNF-α, IL-2, IL-4, IL-6, IL-10 and IL-17 levels were assessed by cytometric bead array (CBA). Results are expressed as mean ± SEM. Asterisk(s) indicate statistical differences, *p <0.05.

**Figure 4:**
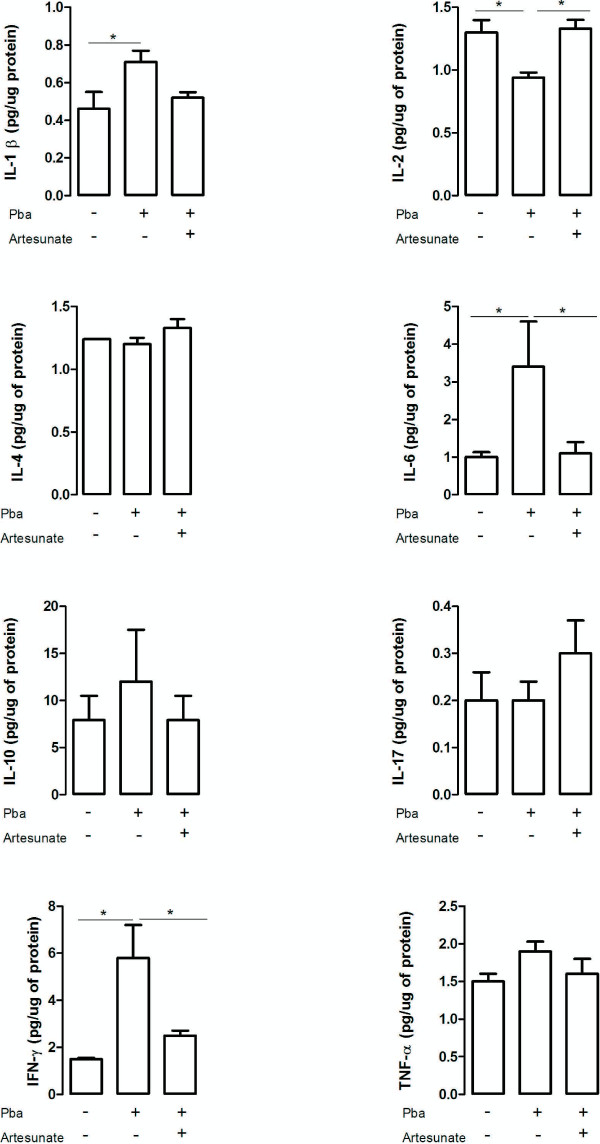
**Cytokine levels in the hippocampus of controls and infected mice following artesunate treatment.** C57BL/6 mice were intraperitoneally infected with 10^6^ parasitized erythrocytes and control (non-infected) animals received the same volume of PBS (n = 5 *per group*). On day 4 post-infection *P .berghei*-infected mice received a single dose of artesunate (ip 32 mg/kg). The hippocampus was collected on day 5 post-infection, homogenized and IL-1β expression was measured by ELISA while IFN-γ, TNF, IL-2, IL-4, IL-6, IL-10 and IL-17 levels were assessed by cytometric bead array (CBA). Results are expressed as mean ± SEM. Asterisk(s) indicate statistical differences where *p < 0.05.

Regarding protein levels, a significant increase of TNF, IL-1β and IFN-γ was found in the frontal cortex of infected animals, whereas a reduction of IL-10 was also observed (Figure 2). In the hippocampus higher levels of IL-1β, IL-6 and IFN-γ were found on day 5 pi in parallel with a decrease in IL-2 expression (Figure 3). No significant differences were detected in the levels of IL-2, IL-4, IL-6 and IL-17 in the frontal cortex, and in the levels of IL-4, IL-10, IL-17 and TNF in the hippocampus when comparing controls and *P. berghei*-infected animals (Figures 3 and 4).

### A single dose of artesunate reduced cytokines levels in the hippocampus and frontal cortex of *P. berghei*-infected mice

The putative anti-inflammatory property of artesunate has been demonstrated in different conditions [17,18]. To investigate its effects in CM, the cytokine levels in frontal cortex, hippocampus and serum of controls and *P. berghei*-infected mice that received or not a single dose of artesunate were assessed.

**Figure 5:**
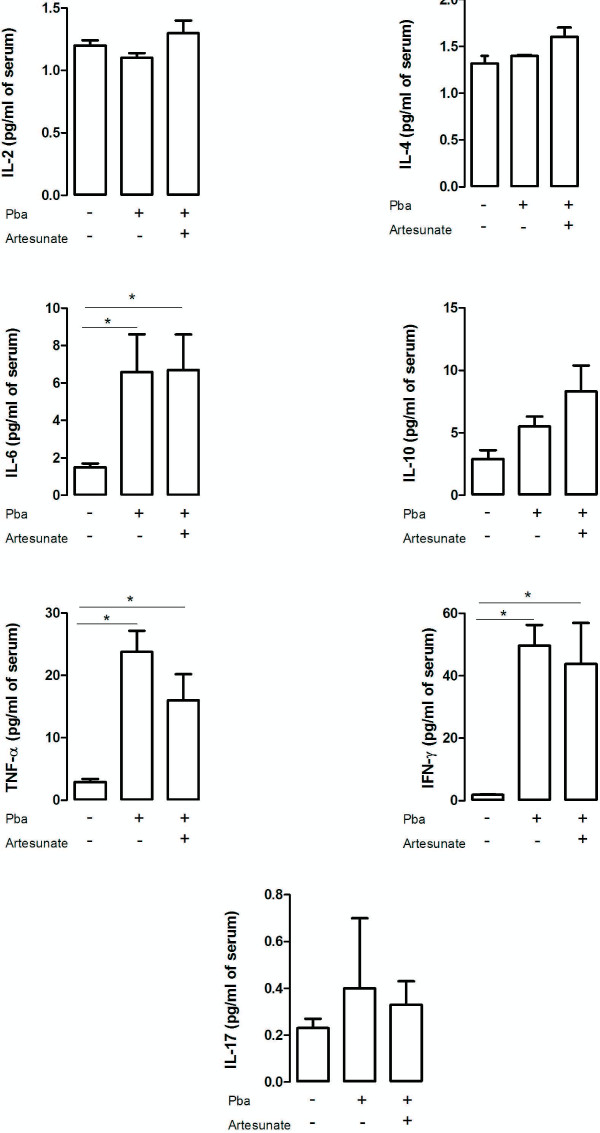
**Cytokine levels in the serum of controls and infected mice following artesunate treatment.** C57BL/6 mice were intraperitoneally infected with 10^6^ parasitized erythrocytes and control (non-infected) animals received the same volume of PBS (n = 5 *per group*). On day 4 post-infection *P. berghei*-infected mice received a single dose of artesunate (ip 32 mg/kg). The serum was collected at day 5 post-infection and IFN-γ, TNF, IL-2, IL-4, IL-6, IL-10 and IL-17 levels were assessed by cytometric bead array (CBA). Results are expressed as mean ± SEM. Asterisk(s) indicate statistical differences. *p < 0.05.

PbA-infected mice presented an increase in frontal cortex levels of TNF (F_(2,12)_ = 3.8; p < 0.05), IFN-γ (F_(2,12)_ = 7.1; p < 0.01) and IL-1β (F_(2,12)=_ 3.6; p < 0.05), and decreased levels of IL-10 (F_(2,12)_ = 3.1; p < 0.05) in comparison with controls (Figure 3). The levels of cytokines of infected mice treated with artesunate in the frontal cortex were similar to controls (Figure 3). *Plasmodium berghei*-infected mice showed an increase in hipoccampal levels of IFN-γ (F_(2,12)_ = 7.7; p < 0.01) and IL-6 (F_(2,12)_ = 5.91; p < 0.05), and decreased levels of IL-2 (F_(2,12)_ = 8.6; p < 0.01) in comparison with controls and treated *P. berghei*-infected mice (Figure 4). No significant difference was found in serum cytokine levels between treated and non-treated *P. berghei*-infected groups. When compared to the controls they exhibited higher serum levels of IL-6 (χ2(df = 2, n = 15) = 9.5, p < 0.05, control *versus P. berghei*, U = 0; z = -2.6,p < 0.05; control *versus P. berghei* + artesunate U = 0; z = 2.,6; p < 0.05), IFN-γ (χ2(df = 2, n = 15) = 10.5, p < 0.05, control *versus P. berghei*, U = 0; z = -2.6,p < 0.05; control *versus P. berghei* + artesunate U = 0;z = 2.,6, p < 0.05) and TNF (χ2(df = 2, n = 15) =10.8, p < 0.05, control *versus P. berghe*, U = 0; z = -2.6, p < 0.05; control *versus P. berghei* + artesunate U = 0; z = 2.6, p < 0.05) than controls (Figure 5).

### A single dose of artesunate improved survival and clinical signs of cerebral malaria

**Figure 6:**
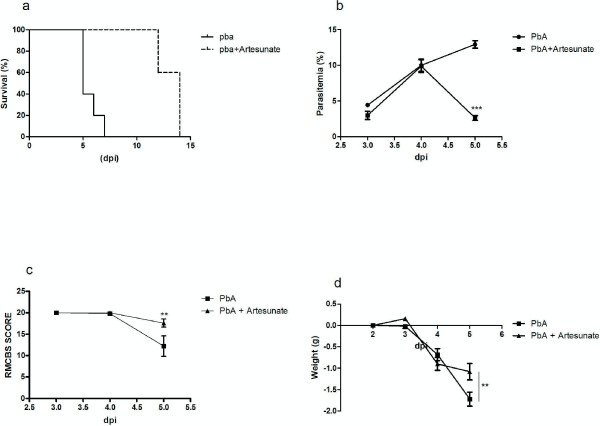
**A single dose of artesunate improved survival and clinical signs of cerebral malaria. (a)** Survival curve comparisons of *P. berghei*-infected mice treated or not treated with artesunate were expressed days after infection; **(b)** natural course of *P. berghei* infection was determined by flow cytometric analysis; **(c)** clinical signs of cerebral malaria assessed by the RMCBS scale; **(d)** weight variation in *P. berghei*-infected mice treated or not treated with artesunate. The data is representative of two independent experiments (n = 5 *per group*) and shown as the mean+/- SEM. Asterisk(s) indicate statistical differences, *p < 0.05.

*Plasmodium berghei* -infected mice treated with a single dose of artesunate presented a significant improvement in survival (χ2 = 8.5, p < 0.001; Figure 6a) and a decrease in the percentage of parasitaemia on day 5 pi when compared with non-treated infected animals (Figure 6b). While *P. berghei*-infected mice presented significant changes in clinical parameters assessed by RMCBS, treated mice did not show clinical symptoms of CM (Figure 6c). Both treated and non-treated *P. berghei*-infected mice lost weight, but it was more significant in non-treated mice at day 5 pi (Figure 6d).

## Discussion

In the present study, short-term aversive memory impairment was associated with increased levels of pro-inflammatory cytokines and decrease of regulatory mediators, such as IL-2 and IL-10, in the hippocampus and frontal cortex of *P. berghei*-infected mice. Therefore, different patterns of cytokines expression seem to be dependent on the region assessed, i e, hippocampus or frontal cortex. This study investigated the expression (mRNA and protein) of cytokines in memory-related areas during CM, and the results reinforce a role for inflammation in the development and persistence of cognitive changes.

Inflammatory processes in the CNS, including an up-regulation of pro-inflammatory cytokines, have been associated with cognitive and behavioural dysfunctions in clinical and experimental CM. For instance, a previous study demonstrated that *P. berghei*-infected mice exhibit anxiety-like symptoms associated with increased brain levels of IL-1β and TNF, and histopathological changes in brainstem, cerebrum and hippocampus [10]. Desruisseaux *et al*. [8] found a significant impairment in working memory of PbA-infected mice, which was associated with cell infiltration and haemorrhage in thalamus, midbrain and cerebellum and with microglial activation in the cortex and hippocampus. Additionally, John *et al*. [27] have suggested a negative correlation between cognitive (attention and working memory) deficits and cerebrospinal fluid (CSF) levels of TNF in children with CM. Although these experimental and human studies have provided evidence for the role of altered levels of cytokines in cognitive and behavioural changes found in CM, few studies investigated the expression of cytokines in specific brain areas, such as hippocampus and frontal cortex, involved in these functions.

A recent study conducted by Linares *et al*. [11] demonstrated an increase in IFN-γ and TNF mRNA expression in several brain areas of *P. berghei*-infected mice, including cortex, hippocampus, thalamus/hypothalamus and brainstem. The upregulation of these cytokines was found in an early or asymptomatic stage of CM and was associated with an increased expression of immunoproteasome subunits. The replacement of proteasome subunits for immunoproteasome factors is a brain-inflammatory response that inhibits normal protein turnover [28]. There is evidence that proteasome also participates in neuronal survival and plasticity [29]. The substitution of proteasome for immunoproteasome complex could lead to neuronal death contributing to the cognitive deficits through impairment of synaptic function [28,30]. The present study provides evidence that pro-inflammatory cytokines expression remain upregulated in the hippocampus and frontal cortex until symptomatic stage of CM, culminating with cognitive impairment. It is worth mentioning that no impairment was found in long-term aversive memory, probably due to significant locomotor and exploratory deficits observed on day 6 pi. In agreement, previous studies have been demonstrated that changes in motor activity during CM occurred on day 6 p.i. whereas anxiety like behaviour appeared earlier on day 5 pi [10,31]. Thus, it seems that behavioural disorders precede locomotor impairment during neuroinflammation caused by *P. berghei* ANKA infection.

CM is a clinical emergence that is invariably fatal without appropriate treatment [12]. Artesunate is a potent anti-malarial drug which has been widely accepted as the treatment of choice for severe malaria [13]. Apart from its well-known antiparasitic property, there is growing evidence suggesting an immunomodulatory activity of artesunate in infectious and autoimmune disorders [14-18]. Artesunate was able to protect mice subjected to sepsis by decreasing serum IL-6 and TNF levels via inhibition of Toll-like receptors expression and NF-κB activation in peritoneal macrophages [17,32,33]. In a rat model of arthritis, artesunate treatment significantly attenuated inflammation and cartilage damage by decreasing the levels of IL-1β, IL-17 and TNF in rat’s hind paws. The anti-arthritic effect of artesunate was associated with NF-κB and mitogen-activated protein kinase signaling pathway suppression [18]. A decrease in cytokine levels and oxidative stress in lung tissue was also found in an allergic asthma model after artesunate treatment [14,15]. Altogether these studies provide strong evidence of artesunate capacity of modulating inflammatory response through the regulation of signaling pathways.

No previous study investigated the effect of artesunate in the CNS inflammation during CM. In the present study, a single dose of artesunate was able to decrease pro-inflammatory cytokines expression in the hippocampus and frontal cortex of *P. berghei*-infected mice in parallel with a significant reduction of parasitaemia and improvement in neurological symptoms and survival.

A single dose of artesunate was highly effective in clearing *P. berghei* parasitaemia at day 5 pi, rescuing mice in the late stages of CM. Reduction of the number of adherent leukocytes in brain vessels of *P. berghei*-infected mice was also observed 24 hours after a single dose of artemether [19]. These authors suggested that the decrease in brain inflammatory response could be a consequence of faster parasitaemia clearance associated with an intrinsic anti-inflammatory property presented by artemisinin derivatives [19]. Interestingly, a systemic release of pro-inflammatory cytokines during the acute phase of malaria is required for the control of parasitaemia [34]. Moreover, the serum levels of cytokines (IL-6, IFN-γ and TNF) in *P. berghei*-infected mice treated with artesunate were comparable to non-treated *P. berghei*-infected animals.

An *in vitro* study conducted by Lee *et al*. [35] showed that artesunate attenuates LPS-induced inflammatory responses in microglial BV2 cells by activating the Nrf2 transcription factor NF-E2-related factor-2 (Nrf2). This transcription factor regulates inflammatory responsiveness in the CNS by mediating the expression of important antioxidant and phase II detoxification genes, and leading to an increase in the level of haem oxygenase-1 [35]. There is evidence that haem oxygenase-1 suppress the pathogenesis of experimental CM by preventing blood-brain barrier disruption, brain microvasculature congestion and neuro-inflammation [36,37]. The current study provided evidence of an anti-inflammatory role of artesunate in the CNS of *P. berghei*-infected mice indicating that CM symptoms prevention was not only due to parasitaemia clearance.

Experimental cerebral malaria development is dependent of Plasmodium strains and mice of susceptible genetic background. In systemic models of malaria, infected mice did not present CNS inflammation as well as CM signs but rather a significant increase in parasitemia and anemia. The lack of cognitive and neurological impairment after parasitemia clearance by chloroquine in those models supports a specific CNS involvement in CM outcome rather than severe systemic disease or the presence of the parasite *per se*[23]. A recent study conducted by Reis *et al*. [38]demonstrated that the statin Lovastatin (which exhibits a significant anti-inflammatory effect) was capable of decreasing pro-inflammatory cytokine levels in CNS during the acute phase of CM without influencing parasitaemia. However, improvement in survival rate was only observed in association with the antimalarial chloroquine. Despite being highly effective in preventing mortality, chloroquine alone did not prevent CM cognitive and behavioral outcome. This indicates that limiting the inflammatory damage to the brain is necessary to improve CM outcome in addition to an effective control of the parasitic disease. *P. berghei* ANKA mice were also treated with sodium diclofenac or allopurinol as control anti-inflammatory agents and did not detect protective effects in CM. In fact, sodium diclofenac increased mortality in infected animals, suggesting that anti-inflammatory drugs could trigger distinct immunomodulatory effects which could be effective or not to improve CM outcome [38].

## Conclusion

In summary, the present study provided further evidence that key areas related to memory, such as hippocampus and frontal cortex, presented different patterns of cytokine expression. Furthermore, it seems that the anti-malarial artesunate presented not only a systemic antiparasitic activity but could also regulate inflammatory responses in the CNS by decreasing local pro-inflammatory cytokines release during the course of CM. As this study is largely descriptive, further studies are required to systematically investigate the immune/inflammatory pathways involved in the anti-inflammatory activity of artesunate in CM as well as its impact on cognitive outcome. Moreover, other symptoms typical of infection conditions such as fever should be also evaluated in association with parasitemia, body weight loss and clinical neurological signs of CM (ataxia, paralysis and coma) to determine the progression of the disease as well as to guide antimalarial treatment.

## Abbreviations

CNS: Central nervous system; CM: Cerebral malaria; CBA: Cytometric bead array; ELISA: Enzyme-linked immunosorbent assay; IFN-γ: Interferon-γ; IL-1β: Interleukin-1 beta; IL-2: Interleukin-2; IL-6: Interleukin-6; IL-10: Interleukin-10; IL-17: Interleukin-17; RMCBS: Rapid murine coma and behaviour scale; SEM: Standard error of the mean; SHIRPA: SmithKline/Harwell/Imperial College/Royal Hospital/Phenotype Assessment; TNF: Tumor necrosis factor; WHO: World health organization

## Competing interests

The authors declare that they have no competing interests.

## Authors’ contributions

ASM participated in the experimental design, carried out behavioural and immunological assays, data analysis and drafted the manuscript. FB participated in the experimental design, data analysis and carried out behavioural and immunological assays. NPR and DHR performed immunological assays. DC and DGS performed real time PCR analysis. FSM and MAR participated in the design and coordination of the study. ALT designed the study and was responsible for the interpretation of experiments and editing the manuscript. ACC participated in the experimental design and in the execution of experiments involving artesunate, immunological assays, data analysis and revised the manuscript. All authors have read and approved the final version of the manuscript.
